# Phase angle as a predictor of prolonged length of hospital stay and adverse outcomes in elderly medical inpatients: a retrospective cohort study

**DOI:** 10.3389/fnut.2025.1623983

**Published:** 2025-08-07

**Authors:** Jia Liu, Song Hu, Shan Wang, Tongxiao Luan, Yuting Duan, Jing Zhou, Li Jia, Nina An, Yongjun Mao

**Affiliations:** ^1^Department of Geriatrics, The Affiliated Hospital of Qingdao University, Qingdao, China; ^2^Department of Geriatrics, Qingdao Medical College, Qingdao University, Qingdao, China

**Keywords:** elderly, malnutrition, bioelectrical impedance, phase angle, adverse outcomes

## Abstract

**Objectives:**

Evaluating prognosis in geriatric inpatients presents significant complexity and challenges. The aim of this retrospective study was to investigate the association between phase angle (PhA) and clinical outcomes in hospitalized elderly patients, specifically focusing on prolonged hospital stays, one-year readmission, or all-cause mortality.

**Methods:**

The study enrolled individuals aged ≥65 years hospitalized in the geriatric medical ward of our hospital. PhA was assessed using BIA, and the length of hospital stay (LOS) was documented. Readmission and mortality outcomes were extracted from electronic medical records and supplemented by telephone follow-ups with patients or their relatives within 1 year following discharge. Optimal PhA thresholds for out-come prediction were determined using Receiver Operating Characteristic curve (ROC). Multivariable Cox proportional hazards regression was employed to evaluate the relationship between PhA and the composite endpoint of readmission or mortality, whereas logistic regression examined its association with LOS.

**Results:**

This study enrolled a total of 218 geriatric patients over a median follow-up duration of 1 year. Among these participants, 42 patients (19.3%) experienced composite endpoint events, defined as either one-year readmission or all-cause mortality. Adverse event rates across the PhA tertiles (T1–T3) were 32.9%, 18.6%, and 5.6%, respectively, indicating a significant decrease in event incidence as PhA values increased. Multivariable-adjusted Cox regression analysis revealed that low PhA was significantly associated with a higher risk of one-year composite endpoint events (HR = 3.657, 95% CI: 1.625–8.229). Additionally, patients with low PhA based on the optimal ROC-derived cutoff had 3.243 times higher odds of prolonged LOS (95% CI: 1.146–9.177).

**Conclusion:**

Low PhA is independently associated with prolonged LOS and higher one-year adverse outcomes in elderly medical inpatients. PhA can serve as a valuable indicator for monitoring malnutrition in hospitalized elderly patients and functions as a reliable independent predictor of prognosis.

## 1 Introduction

The disease burden resulting from population aging has emerged as a major challenge in the fields of biomedical and public health research (1, 2). Older adults aged ≥ 60 years account for 23% of the global disease burden (3). The rising prevalence of chronic diseases and age-related functional decline substantially contributes to higher hospitalization rates and adverse clinical outcomes in the elderly population. A Chinese epidemiological study revealed that the hospitalization rates among adults aged 65 and above have been increasing annually, with a one-year readmission rate as high as 25.27% (4). Poor clinical outcomes among hospitalized elderly patients contribute to increased healthcare expenditures, diminished quality of medical care, and disruptions to the daily lives of both patients and their families (5). Therefore, recognizing risk factors and implementing tailored interventions can potentially enhance quality of life, improve functional capacity, and decrease readmission and mortality rates in this population (6).

Malnutrition is a prevalent geriatric syndrome. Research has demonstrated that over than 50% of hospitalized older adults present with nutritional risk or malnutrition, and this condition may further deteriorate at discharge (7, 8). Nutritional status serves as a crucial role in determining health outcomes, as malnourished individuals face increased hospitalization costs, longer hospital length of stay (LOS), and elevated risks of readmission and mortality (7, 9, 10). Multiple nutritional screening tools are currently available, and body composition analysis has increasingly demonstrated its clinical relevance in the context of nutritional assessment in recent years (11). As a validated, non-invasive tool, bioelectrical impedance analysis (BIA) provides cost-effective body composition analysis with broad clinical applicability across medical specialties (12).

Calculated from BIA-derived resistance and reactance, phase angle (PhA) is a validated measure of cellular health, with lower PhA values indicating impaired cellular integrity or impending cell death (13). This independent parameter serves as a biomarker for evaluating cellular function, hydration status and nutritional condition, exhibiting strong correlations with disease diagnosis and prognostic indicators (14). As evidence has shown, PhA functions as a biomarker for evaluating and monitoring morbidity and mortality in respiratory diseases, and may serve as a critical indicator for predicting the mortality risk of COVID-19 (15, 16). In addition, PhA exhibits negative correlations with muscle mass and function in elderly individuals, potentially serving as a marker for the identification of sarcopenia (17, 18). Lower PhA significantly increases the risk of frailty, disability and adverse outcomes among elderly patients (19–21). Additionally, a separate study suggests that the PhA predicts a poor prognosis in general medical patients (22).

Geriatric patients often present challenges in prognostic evaluation due to complex factors such as multimorbidity and polypharmacy. While existing evidence substantiates the clinical validity of PhA for disease assessment, the predictive capacity of PhA for clinical outcomes among hospitalized elderly adults remains underexplored. To address this knowledge gap, we hypothesized that lower PhA would independently predict the composite endpoint of one-year readmission or all-cause mortality in elderly medical inpatients. Additionally, we examined the influence of PhA on LOS.

## 2 Materials and methods

### 2.1 Study design

**Figure 1:**
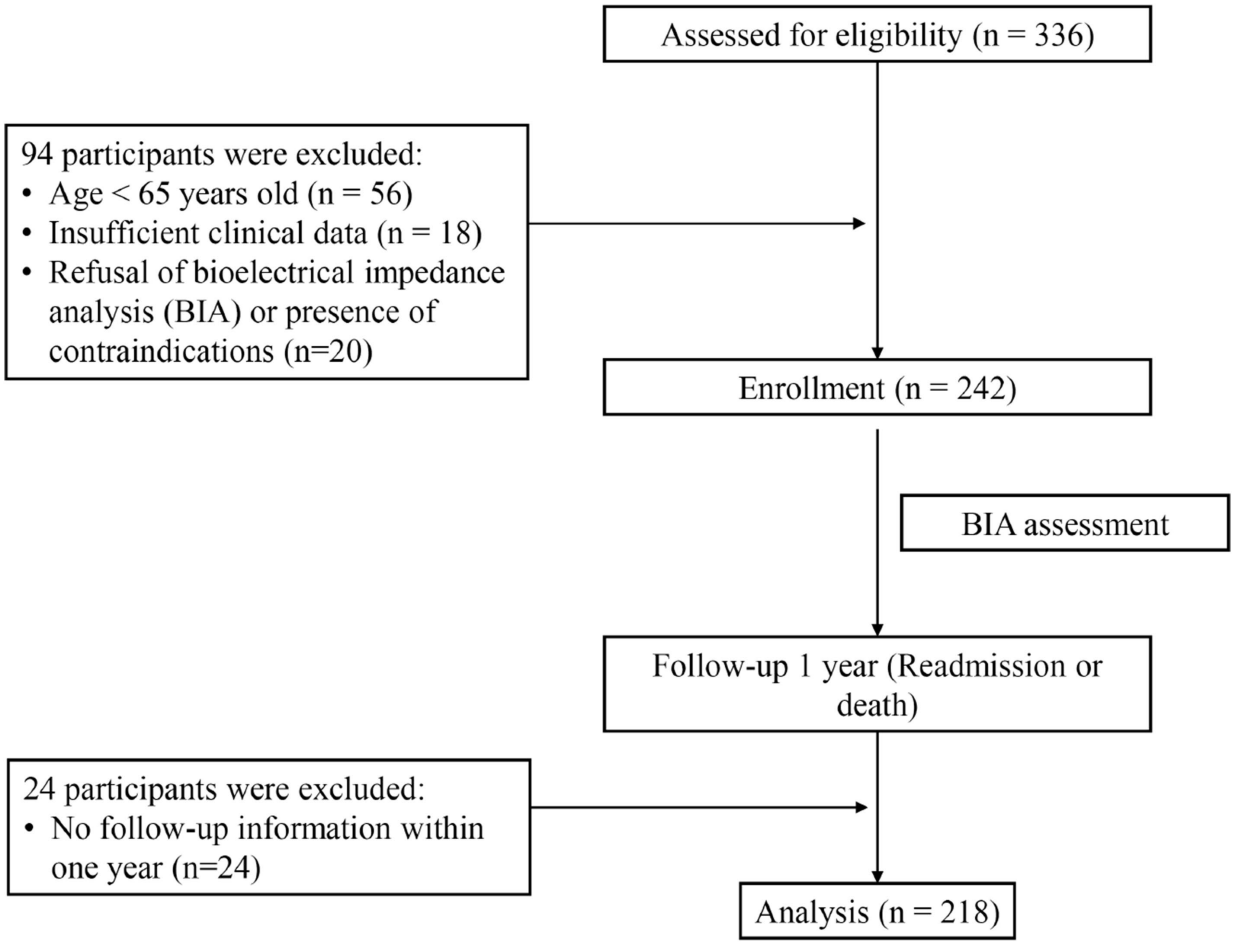
Research flowchart.

The study enrolled patients aged 65 years or older in the geriatric medical ward of the Affiliated Hospital of Qingdao University between July 2019 and January 2023. A total of 336 individuals were initially screened. We excluded patients younger than 65 years, those with incomplete clinical records, individuals who declined or had contraindicated for BIA examination, and cases lost to follow-up. Patients with severe edema were excluded prior to BIA, as severe fluid retention significantly distorts body composition measurements. Finally, a cohort of 218 patients was included in the analysis, with the research flowchart shown in Figure 1.

### 2.2 Assessment of PhA by BIA

The PhA was assessed by BIA using the InBody S10^®^ device (InBody, Biospace Co., Ltd., Seoul, Korea), in accordance with the manufacturer’s protocol and a previously published methodological guideline (23). Parameters recorded included PhA, skeletal muscle mass index (SMI), visceral fat area (VFA), and waist circumference (WC).

### 2.3 Sociodemographic data and laboratory test indicators

Data were collected by well-trained assessors. Sociodemographic data and clinical information were obtained through a systematic review of medical records or structured interviews. Body mass index (BMI) was derived from anthropometrically measured height and weight. All patients underwent blood pressure assessments and routine laboratory examinations. The cardiac and renal functions, as well as the hydration status of the participants, were evaluated through measurements of NT-ProBNP and estimated glomerular filtration rate (eGFR).

### 2.4 Assessment of geriatric syndrome

The medication history, functional status, multimorbidity, and nutritional status of elderly inpatients were evaluated through structured interview questionnaires. Polypharmacy was assigned to patients receiving ≥5 medications concomitantly. Cognitive function was evaluated by the Mini-Mental State Examination (MMSE). Functional dependence was assessed by the 100-point Activities of Daily Living (ADL) scale with established cutoffs: 100 (normal), 61–99 (mild disability), 41–60 (moderate disability), and ≤40 (severe disability). We categorized functional trajectory as either “stable/improved” or “declined/death” by comparing pre-admission (2-week baseline) and pre-discharge ADL scores. Multimorbidity was quantified based on the presence of seven chronic diseases in older inpatients, including coronary heart disease, hyper-tension, diabetes, chronic obstructive pulmonary disease (COPD), cerebrovascular disease, cancer, and chronic kidney disease, as self-reported by patients and confirmed by physician diagnosis. Nutritional assessment was performed via the Geriatric Nutritional Risk Index (GNRI), with a score of ≤98 indicating malnutrition classification (24). The GNRI formula applied was: $\begin{array}{l} \text{GNRI}=(1.489\times \text{serum albumin}(g/L))+\\ \;\;(41.7\times \text{current weight}\;(\mathrm{kg})/\text{ideal weight}\;(\mathrm{kg})). \end{array}$ Sex-specific ideal weights were calculated as follows: for men, ideal weight=height(cm)−100−[(height(cm)−150)/4] , while for women it was calculated as: ideal weight=height(cm)−100−[(height(cm)−150)/2.5] (24).

### 2.5 Endpoints

With a median observation period of 1 year, we examined a composite adverse outcome that included all-cause mortality and unplanned readmission occurring within 12 months after discharge. Follow-up events were determined by reviewing electronic medical records or telephone interview records. During follow-up, the period spanning from initial hospitalization to the occurrence of the first reported adverse event was measured to determine the timing of the composite adverse outcome. For participants who did not experience any adverse events, follow-up duration reflected the period from initial assessment to last available survey date. Elderly patients were categorized based on the interquartile range (IQR) of hospitalization duration into two groups: prolonged stay (upper IQR) and normal stay, in order to evaluate the association between PhA and LOS.

### 2.6 Statistical analysis

Normality testing was performed using the Shapiro–Wilk test. Continuous variables were presented as mean ± standard deviation (SD) for normally distributed data or median (Q_1_–Q_3_) for non-normally distributed data, according to the normality test results. Categorical variables were presented as numbers and percentages [*n*, (%)], and were compared using chi-square tests or Fisher ‘s exact test, as appropriate. Group comparisons used t-tests/ANOVA for normally distributed data and Mann–Whitney *U*/Kruskal–Wallis tests for non-normal distributions. Bivariate correlations were assessed using Pearson’s or Spearman’s correlation coefficients.

Kaplan–Meier analysis was performed to evaluate the one-year cumulative incidence of composite adverse outcomes among elderly patients categorized by PhA tertiles. Survival curves were compared using the log-rank test. Receiver operating characteristic curve (ROC) analysis was utilized to establish the optimal PhA threshold for one-year composite outcomes and LOS prediction, with the area under the curve (AUC) serving as a metric for quantifying predictive accuracy. Multivariate Cox regression analysis was utilized to quantify the relationship between PhA and the risk of composite adverse outcome. To assess the proportional hazards assumption, Schoenfeld residuals tests were performed, demonstrating no significant deviations (all *p* > 0.05; Supplementary Table S1). Multivariate logistic regression models were used to evaluated the relationship between PhA and LOS. Variance inflation factor (VIF) analysis was conducted to evaluate potential multicollinearity and confirmed no significant multicollinearity among the adjusted variables (all VIF < 5; Supplementary Table S2). Additionally, bootstrapping methods were employed in sensitivity analyses to validate the consistency of the results. A *p*-value < 0.05 was considered statistically significant. Data analysis was performed with SPSS 26.0, R 4.5.1 and Origin 2024 software.

## 3 Results

### 3.1 Baseline characteristics

The study enrolled 218 elderly inpatients with a median age of 75.50 (69.00, 81.00), of whom 55.05% were male. The mean PhA was 4.73 ± 0.93°. Participants were stratified into tertiles (T) based on PhA: low PhA (T1, ≤4.4°), medium PhA (T2, 4.4°–5.1°), and high PhA (T3, >5.1°). Baseline characteristics of the patients were presented in Table 1. Participants in the lowest PhA tertile were significantly older and exhibited a higher prevalence of polypharmacy, malnutrition, and disability (all *p* < 0.001). This group also demonstrated elevated NT-proBNP levels, along with reduced albumin, prealbumin, BMI, and SMI (all *p* < 0.001). Notably, these participants showed a declining functional trajectory and lower eGFR (all *p* < 0.05).

**Table 1:** Population baseline characteristics by PhA tertiles.

Variables	Total (*n* = 218)	PhA tertiles			*P*-value
		T1 (*n* = 76)	T2 (*n* = 70)	T3 (*n* = 72)	
Age, years, M (Q₁, Q₃)	75.50 (69.00, 81.00)	80.50 (76.00, 85.00)	74.50 (68.75, 80.00)	70.00 (67.00, 74.00)	<0.001
Male, *n* (%)	120 (55.05)	39 (51.32)	29 (41.43)	52 (72.22)	<0.001
Education, *n* (%)					0.965
Junior high school or lower	76 (34.86)	28 (36.84)	23 (32.86)	25 (34.72)	
High school or vocational high school	83 (38.07)	27 (35.53)	29 (41.43)	27 (37.50)	
Associate degree or higher	59 (27.06)	21 (27.63)	18 (25.71)	20 (27.78)	
Residence, *n* (%)					0.461
Rural	46 (21.10)	19 (25.00)	15 (21.43)	12 (16.67)	
Urban	172 (78.90)	57 (75.00)	55 (78.57)	60 (83.33)	
Marriage, *n* (%)					<0.001
Married	156 (71.56)	41 (53.95)	55 (78.57)	60 (83.33)	
Others	62 (28.44)	35 (46.05)	15 (21.43)	12 (16.67)	
Health Insurance, *n* (%)					0.461
Employment-based Health Insurance	172 (78.90)	57 (75.00)	55 (78.57)	60 (83.33)	
Non-employment-based Health Insurance	46 (21.10)	19 (25.00)	15 (21.43)	12 (16.67)	
Cognitive impairment, *n* (%)	29 (13.30)	14 (18.42)	9 (12.86)	6 (8.33)	0.194
Smoking, n (%)	29 (13.3)	14 (18.4)	5 (7.1)	10 (13.9)	0.132
Drinking, *n* (%)	25 (11.5)	10 (13.2)	3 (4.3)	12 (16.7)	0.058
Multimorbidity, M (Q₁, Q₃)	2.00 (1.00, 3.00)	2.00 (1.00, 3.00)	2.00 (1.00, 3.00)	2.00 (1.00, 3.00)	0.879
Polypharmacy, *n* (%)	107 (49.1)	50 (65.8)	38 (54.3)	19 (26.4)	<0.001
Malnutrition, *n* (%)	126 (57.8)	59 (77.6)	35 (27.8)	32 (25.4)	<0.001
Functional trajectory, *n* (%)					0.003
Stable/Improved	189 (86.70)	58 (76.32)	63 (90.00)	68 (94.44)	
Decline/Death	29 (13.30)	18 (23.68)	7 (10.00)	4 (5.56)	
Disability, *n* (%)					<0.001
Normal	109 (50.00)	24 (31.58)	41 (58.57)	44 (61.11)	
Mild	78 (35.78)	30 (39.47)	21 (30.00)	27 (37.50)	
Moderate	18 (8.26)	12 (15.79)	5 (7.14)	1 (1.39)	
Severe	13 (5.96)	10 (13.16)	3 (4.29)	0 (0.00)	
SBP, mmHg, Mean ± SD	136.57 ± 17.6	138.63 ± 18.58	136.07 ± 17.25	134.88 ± 17.61	0.606
DBP, mmHg, Mean ± SD	73.35 ± 10.43	71.91 ± 12.09	74.46 ± 9.28	73.81 ± 9.54	0.081
FBG, mmol/L, M (Q₁, Q₃)	5.16 (4.70, 6.24)	4.98 (4.50, 6.13)	5.36 (4.76, 6.47)	5.13 (4.80, 6.19)	0.099
Albumin, g/L, M (Q₁, Q₃)	37.35 (34.90, 39.60)	35.60 (32.23, 37.95)	38.05 (35.70, 39.85)	38.30 (36.35, 39.90)	<0.001
Prealbumin, mg/L, M (Q₁, Q₃)	225.35 (183.93, 262.60)	210.75 (166.37, 241.85)	224.10 (201.00, 264.75)	240.60 (212.50, 267.75)	<0.001
eGFR, ml/min/1.73m^2^, M (Q₁, Q₃)	70.94 (60.16, 78.91)	68.03 (55.53, 77.69)	71.43 (64.14, 78.91)	72.34 (64.05, 79.71)	0.035
NT-proBNP, (pg/mL), M (Q₁, Q₃)	108.00 (60.00, 236.00)	255.50 (125.00,681.00)	105.50 (66.50,137.50)	58.00 (39.00,100.00)	<0.001
TC, mmol/L, Mean ± SD	4.43 ± 1.14	4.30 ± 1.15	4.62 ± 1.20	4.39 ± 1.06	0.215
TG, mmol/L, M (Q₁, Q₃)	1.08 (0.81, 1.61)	1.08 (0.78, 1.65)	1.08 (0.83, 1.61)	1.09 (0.83, 1.45)	0.936
LDL-C, mmol/L, Mean ± SD	2.41 ± 0.90	2.36 ± 0.98	2.52 ± 0.86	2.34 ± 0.86	0.456
HDL-C, mmol/L, M (Q₁, Q₃)	1.37 (1.16, 1.60)	1.33 (1.13, 1.61)	1.40 (1.20, 1.62)	1.35 (1.16, 1.55)	0.482
BMI, kg/m^2^, Mean ± SD	24.44 ± 3.27	22.93 ± 3.36	24.71 ± 3.05	25.77 ± 2.73	<0.001
SMI, kg/m^2^, Mean ± SD	7.60 ± 1.18	7.06 ± 1.20	7.44 ± 1.06	8.33 ± 0.87	<0.001
WC, cm, Mean ± SD	80.10 ± 10.52	78.13 ± 12.35	80.51 ± 10.22	81.80 ± 8.27	0.097
VFA, cm^2^, M (Q₁, Q₃)	77.75 (57.18, 104.15)	77.60 (54.45, 111.50)	80.05 (59.40, 116.80)	71.30 (55.58, 88.10)	0.113
PhA, °, Mean ± SD	4.73 ± 0.93	3.74 ± 0.59	4.80 ± 0.20	5.73 ± 0.44	<0.001

BMI, body mass index; DBP, diastolic blood pressure; eGFR, estimated glomerular filtration rate; FBG, fasting blood glucose; HDL-C, high-density lipoprotein cholesterol; LDL-C, low-density lipoprotein cholesterol; PhA, phase angle; SBP, systolic blood pressure; SMI, skeletal muscle mass index; TC, total cholesterol; TG, triglycerides; VFA, visceral fat area; WC, waist circumference. M, median; Q₁, 1st quartile; Q₃, 3st quartile; SD, standard deviation; T, tertiles.

### 3.2 The correlation between PhA and in-hospital prognosis risk factors

**Figure 2:**
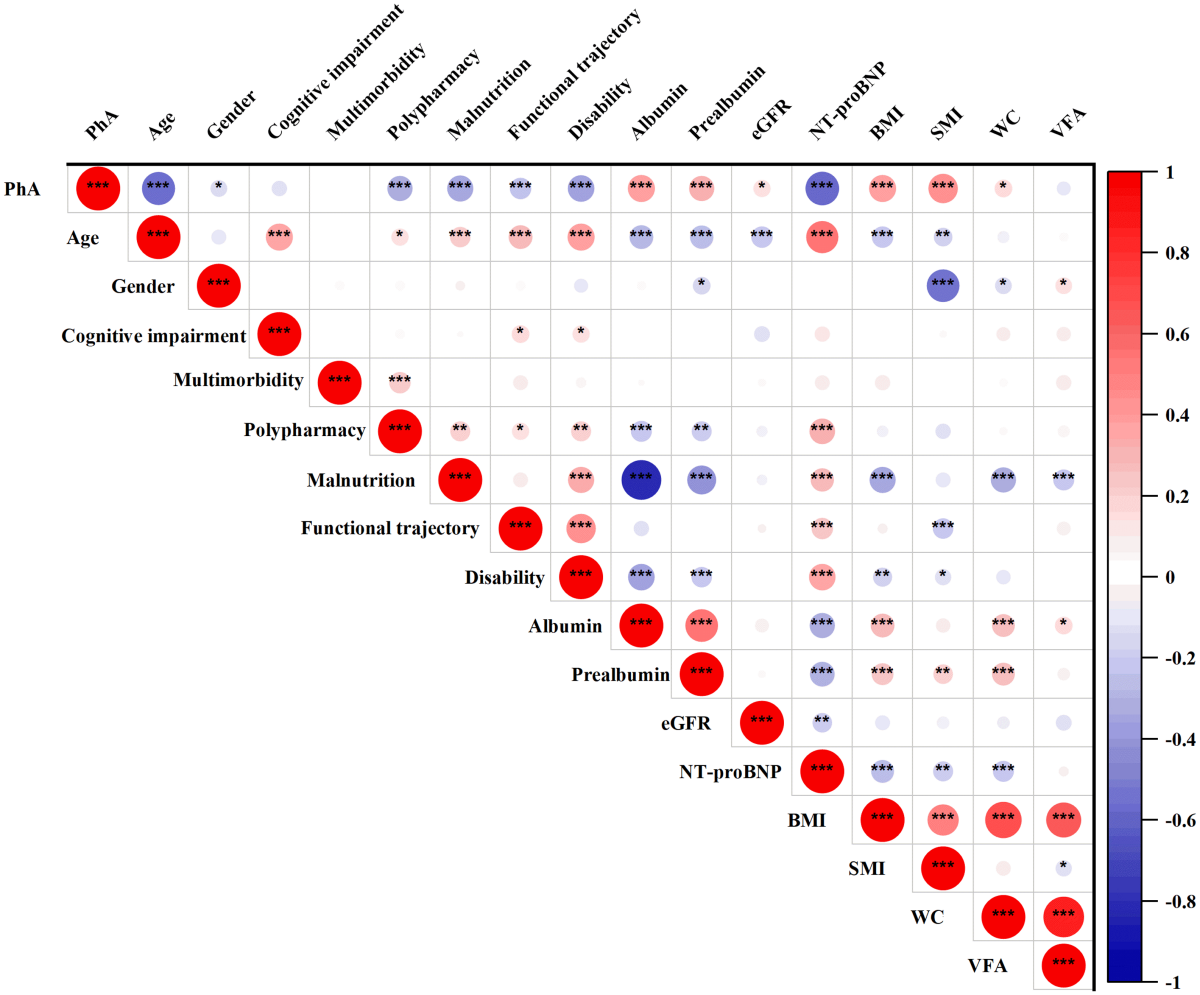
Correlation between PhA and prognostic risk factors in geriatric inpatient. The positive correlation is represented in red, while the negative correlation is depicted in blue. The correlation coefficient ranges from −1 to +1; a higher absolute value of correlation corresponds to a larger circle. BMI, Body Mass Index; eGFR, Estimated Glomerular Filtration Rate; PhA, Phase Angle; SMI, Skeletal Muscle Mass Index; VFA, Visceral Fat Area; WC, Waist Circumference. Significant level: ^*^p < 0.05, ^**^p < 0.01, ^***^p < 0.001.

Figure 2 illustrated the relationship between PhA and various prognostic risk factors in elderly medical inpatients. The results revealed a significant positive correlation between PhA and albumin (0.448, *p* < 0.001), prealbumin (0.387, *p* < 0.001), eGFR (0.156, *p* < 0.05), BMI (0.388, *p* < 0.001), and SMI (0.451, *p* < 0.001). Conversely, it exhibited significant negative correlations with age (−0.585, *p* < 0.001), polypharmacy (−0.333, *p* < 0.001), malnutrition (−0.361, *p* < 0.001), functional trajectory (−0.241, *p* < 0.001), disability (−0.449, *p* < 0.001) and NT-proBNP (−0.595, *p* < 0.001).

### 3.3 PhA and one-year composite adverse outcomes

**Figure 3:**
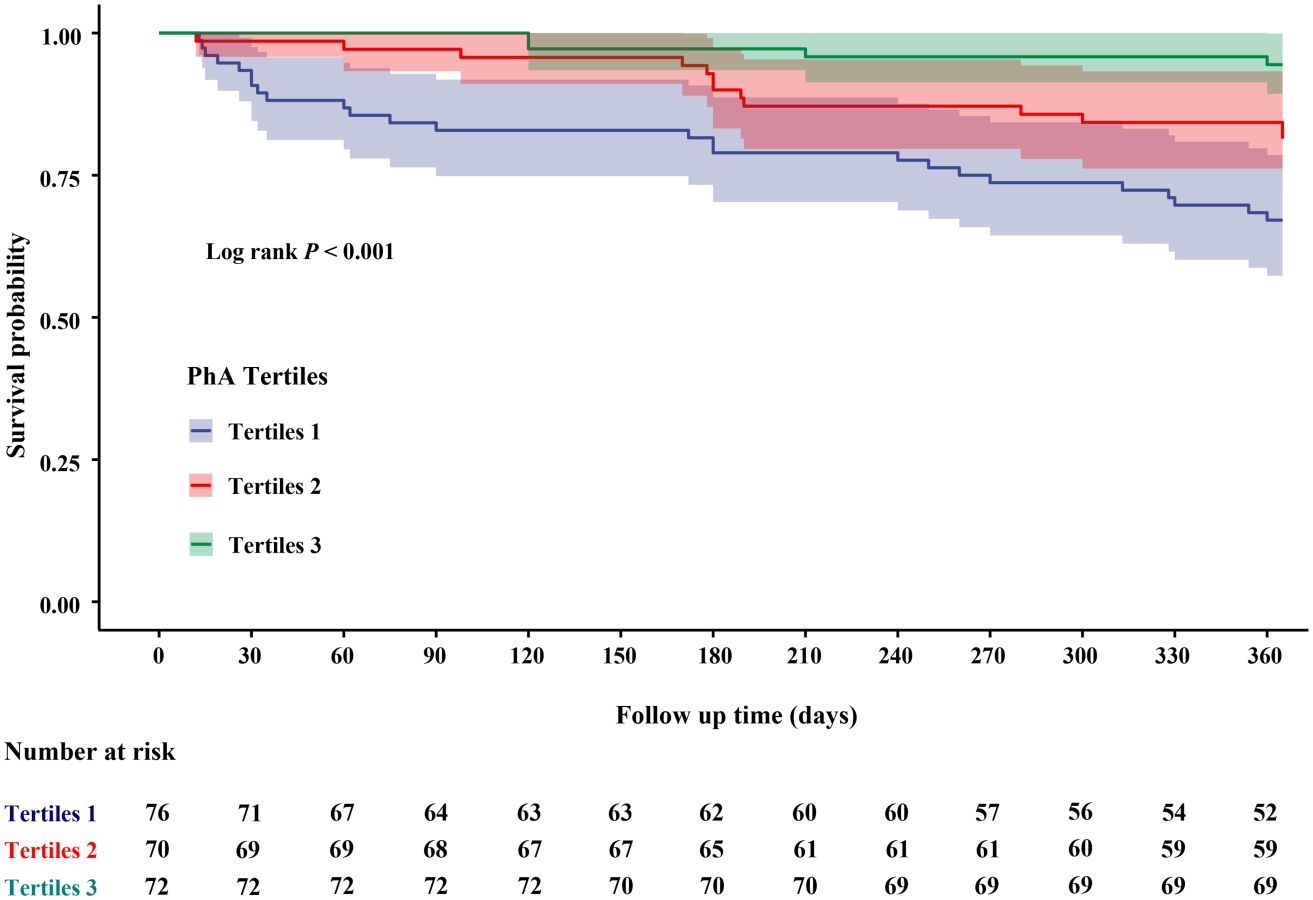
Kaplan–Meier plot of PhA and composite adverse outcomes within 1 year. PhA, Phase Angle.

During the one-year follow-up period, 42 individuals (19.3%) experienced composite endpoint events (Supplementary Table S3). Patients who experienced composite adverse outcomes were significantly older, exhibited lower levels of serum albumin and HDL-C (*p* < 0.05), demonstrated a higher prevalence of disability and polypharmacy, as well as significantly higher NT-proBNP and lower PhA values (*p* < 0.001), relative to those without adverse outcomes. Kaplan–Meier survival curve was presented in Figure 3. Within one-year, elderly participants in the lowest one-third PhA group exhibited a significantly higher incidence of composite adverse outcomes, with cumulative incidences of 32.9% for T1, 18.6% for T2, and 5.6% for T3 (log-rank *p* < 0.001). Generally, a lower PhA was associated with an increased risk of composite adverse outcomes during the follow-up period.

### 3.4 The predictive value of PhA for composite adverse outcomes within 1 year

**Figure 4:**
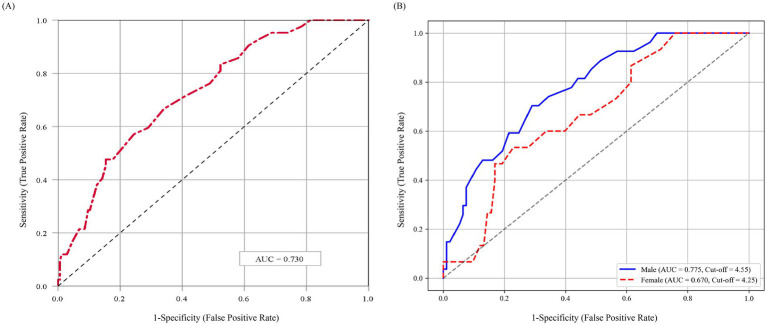
ROC curve of PhA for predicting composite adverse outcomes. **(A)** ROC analysis based on the overall study population. **(B)** Sex-specific ROC analyses. AUC, area under the Curve; PhA, phase angle. The PhA cutoff values were 4.55° for men and 4.25° for women.

The ROC analysis assessed the predictive performance of PhA for composite adverse outcomes within 1 year for elderly hospitalized patients. As shown in Figure 4A, PhA exhibited moderate predictive accuracy, with an AUC of 0.730 (95% confidence interval [CI]: 0.650–0.809), indicating clinically meaningful discriminatory capacity. Furthermore, subgroup analyses stratified by gender revealed that PhA remained a significant predictor of one-year composite adverse outcomes for both male (AUC = 0.775) and female (AUC = 0.670) participants (Figure 4B). The optimal cut-off values were identified as 4.55° for males and 4.25° for females, respectively.

### 3.5 The relationship between PhA dichotomized by cutoff value and prognosis of elderly medical inpatients

Based on sex-specific optimal cutoff values of PhA, the study population was categorized into low-PhA and normal-PhA groups. A total of 73 participants (33.5%) were classified into the low-PhA group. Comparative analyses of clinical characteristics between these two groups were presented in Supplementary Table S4.

Cox proportional hazards regression was used to evaluate the association between PhA and one-year composite adverse outcomes (Table 2). The unadjusted model showed that the low PhA group had a 4.371-fold increased risk of composite adverse outcomes (hazards ratio [HR] = 4.371, 95% CI: 2.324–8.224). After adjusting for geriatric prognostic factors and fluid status in multivariate models, this association remained significant. The fully adjusted model (Model 3) demonstrated that the low PhA group maintained a 3.657-fold higher risk (adjusted HR = 3.657, 95% CI: 1.625–8.229). When analyzed as a continuous variable, each 1-degree increase in PhA was associated with a 66.1% reduction in adverse outcome risk (adjusted HR = 0.339, 95% CI: 0.207–0.555). Sensitivity analysis using 1,000 bootstrap resamples yielded consistent results, showing an average 56.1% risk reduction per 1° PhA increase (mean HR = 0.439, 95% CI: 0.309–0.578).

**Table 2:** Relationship between PhA and composite adverse outcome.

PhA categories	Model 1		Model 2		Model 3	
	HR (95% CI)	*P*-value	HR (95% CI)	*P*-value	HR (95% CI)	*P*-value
PhA (Per 1° increase)	0.439 (0.320, 0.601)	<0.001	0.410 (0.278, 0.602)	<0.001	0.339 (0.207, 0.555)	<0.001
Normal PhA	Reference		Reference		Reference	
Low PhA	4.371 (2.324, 8.224)	<0.001	4.040 (1.933, 8.445)	<0.001	3.657 (1.625, 8.229)	0.002

Model 1: Unadjusted model.

Model 2: Adjusted for age and gender.

Model 3: Model 2 plus polypharmacy, functional trajectory, disability, malnutrition, eGFR, NT-proBNP.

eGFR, estimated glomerular filtration rate; PhA, phase angle. CI, confidence interval; HR, Hazards Ratio.

### 3.6 The relationship between PhA and prolonged LOS

The study examined the association between PhA and LOS. ROC analysis demonstrated that PhA effectively predicted prolonged LOS, with an AUC of 0.774 (95% CI: 0.698–0.850). The optimal PhA cutoffs for identifying prolonged hospitalization were 4.45° for males and 4.25° for females (Supplementary Figure S1). Logistic regression analysis further assessed this association (Table 3). In the unadjusted model, each 1° increase in PhA was associated with a 72.6% decrease in the risk of prolonged LOS (odds ratio [OR] = 0.274, 95% CI: 0.173–0.434). This protective association maintained statistical significance after adjustment for additional risk factors (adjusted OR = 0.419, 95% CI: 0.216–0.814). In contrast, ROC-based stratification revealed that patients with low PhA exhibited a 3.243-fold higher risk of prolonged hospitalization (adjusted OR = 3.243, 95% CI: 1.146–9.177). The bootstrap analysis with 1,000 resamples provided robust validation, indicating approximately a 70% decrease in prolonged LOS risk for every 1° increment in PhA (mean OR = 0.293, 95% CI: 0.187–0.422). These consistent findings underscore PhA’s role as an independent protective factor against extended hospital stays.

**Table 3:** Relationship between PhA and prolonged LOS.

PhA categories	Model 1		Model 2		Model 3	
	OR (95% CI)	*P*-value	OR (95% CI)	*P*-value	OR (95% CI)	*P*-value
PhA (Per 1°increase)	0.274 (0.173, 0.434)	<0.001	0.291 (0.170, 0.499)	<0.001	0.419 (0.216, 0.814)	0.010
Normal PhA	Reference		Reference		Reference	
Low PhA	7.611 (3.703, 15.642)	<0.001	5.411 (2.413, 12.132)	<0.001	3.243 (1.146, 9.177)	0.027

Model 1: Unadjusted model.

Model 2: Adjusted for age and gender.

Model 3: Model 2 plus polypharmacy, functional trajectory, disability, albumin, eGFR.

eGFR, Estimated Glomerular Filtration Rate; PhA, Phase Angle. CI, Confidence Interval; OR, Odds Ratio.

## 4 Discussion

Elderly hospitalized patients often exhibit complex multimorbidity, yet sensitive prognostic markers for this population remain lacking. PhA has recently emerged as a promising prognostic indicator, with growing evidence supporting its clinical significance (14, 25–27). While some studies have explored PhA in clinical populations, its relationship with outcomes in geriatric medical inpatients has not been extensively investigated. Therefore, this study aims to specifically focuses on the predictive value of PhA for adverse outcomes following admission to geriatric medical wards. Our findings demonstrated that lower PhA is an independent predictor for one-year adverse outcomes and is significantly associated with prolonged LOS in geriatric patients.

Substantial evidence indicates that nutritional impairments elevate the risk of adverse clinical outcomes, negatively impact disease progression and recovery, extend hospitalization durations, and lead to functional decline or mortality (28, 29). Researches had confirmed the utility of PhA in the early identification of malnutrition among geriatric patients with multiple chronic conditions (30). In older patients with subacute stroke, a PhA below 4.08° predicted high nutritional risk (31). Additionally, studies have established PhA as an independent predictor of malnutrition and sarcopenia in elderly COPD individuals (32). In this study, we focused on the associations between PhA and nutritional status, the overall prevalence of malnutrition, as defined by the GNRI score, was 57.8%, which was in good agreement with previous reports by Hongyuan et al. (7). Significantly, the prevalence of malnutrition in the lowest tertile of the PhA group reached as high as 77.6%.

Numerous studies have consistently demonstrated the prognostic significance of PhA across a range of clinical outcomes. A prospective study indicated a significant association between reduced PhA and impaired ADL function in hemodialysis patients (33). Another cohort study revealed an independent association between low PhA and all-cause mortality within 1 year in ICU patients (34). In this study, we evaluated the predictive ability of PhA for adverse outcomes in geriatric inpatients using ROC curve analysis. The results demonstrated that PhA had moderate predictive value for one-year composite adverse outcomes (AUC = 0.730). The sex-specific optimal PhA cutoffs were 4.55° for males and 4.25° for females. Currently, no consensus exists on PhA reference values, limiting comparisons with broader populations. Previous studies have reported PhA cutoffs of 5.04° (males) and 4.20° (females) for predicting sarcopenia in older adults (18), while another study identified a PhA < 4.0° as an independent predictor of 90-day readmission or mortality in acute heart failure patients (35). In older hip fracture patients, sex-specific PhA cutoffs for 12-month mortality were 4.05° (females) and 4.65° (males) (36). The PhA cutoff ranges observed in our study align closely with previously reported values, however, further research is still required to establish universally standardized thresholds. To quantify the association between PhA and adverse outcomes, we performed a Cox regression analysis. After adjusting for covariates, each 1° increase in PhA was associated with a 66.1% decrease in the risk of one-year readmission or mortality. Similarly, low PhA based on sex-specific ROC-derived cutoffs significantly increased this risk (adjusted HR = 3.657, 95% CI: 1.625–8.229). This association may be attributed to PhA reflecting malnutrition, decreased skeletal muscle mass, impaired cellular health, and underlying oxidative stress or inflammatory damage (37, 38). Our findings support PhA as a valuable prognostic indicator for readmission and mortality in geriatric inpatients.

The LOS is a reliable indicator of healthcare quality, impacting both medical burden and disease prognosis. Previous studies have consistently shown a significant relationship between malnutrition and prolonged LOS (39, 40). A study focusing on critically ill ICU patients revealed that low PhA accurately discriminated nutritionally high-risk cases and was associated with more than double the ICU stay duration (41). Our findings similarly indicate that, after adjusting for confounding factors, low PhA increased the risk of prolonged LOS in elderly patients. However, it is important to note that prolonged LOS definitions vary widely and may be influenced by non-medical determinants, making it challenging to account for all potential confounding factors in clinical research. Therefore, this association should be interpreted with caution. Nevertheless, PhA remains a relatively effective and convenient tool for evaluating prolonged LOS in elderly hospitalized patients.

Several limitations should be acknowledged. First, the single-center retrospective design and reliance on BIA-measured participants may limit generalizability and introduce selection bias. Second, although sex-specific PhA cutoffs were derived, potential overfitting remains a concern due to the lack of external validation; further analyses would have strengthened causal inferences and enhanced the reliability of the findings. In addition, variations in BIA devices complicate cross-study comparisons. Third, although multivariate adjustments and sensitivity analyses were applied, unmeasured confounders could persist due to the observational nature of this study. Future multicenter prospective studies are needed to validate PhA’s prognostic utility and explore its mechanistic links with clinical outcomes.

In conclusion, this study demonstrated that PhA derived from BIA serves as a valuable predictive biomarker for clinical outcomes in elderly medical inpatients, independently predicting prolonged LOS and the risk of one-year readmission or all-cause death. As a non-invasive measurement, it does not require additional physical effort and serves as an elder-friendly assessment tool that is particularly well-suited for elderly patients and geriatric medical care settings.

## 5 Conclusion

In summary, elderly hospitalized patients exhibited a high incidence of adverse outcomes within 1 year, and PhA appears to be a promising independent predictor. Actively incorporating PhA into routine evaluations in geriatric medical wards may facilitate the identification of the most vulnerable patients, thereby enabling the provision of enhanced care and support.

## Data Availability

The original contributions presented in the study are included in the article/Supplementary material, further inquiries can be directed to the corresponding author.

## Glossary

ADL: Activities of Daily Living
AUC: Area Under the Curve
BIA: Bioelectrical Impedance Analysis
BMI: Body Mass Index
COPD: Chronic Obstructive Pulmonary Disease
CI: Confidence Interval
DBP: Diastolic Blood Pressure
eGFR: Estimated Glomerular Filtration Rate
FBG: Fasting Blood Glucose
GNRI: Geriatric Nutritional Risk Index
HDL-C: High-Density Lipoprotein Cholesterol
HR: Hazards Ratio
IQR: Interquartile Range
LOS: Length of Hospital Stay
LDL-C: Low-Density Lipoprotein Cholesterol
MMSE: Mini-Mental State Examination
OR: Odds Ratio
PhA: Phase Angle
ROC: Receiver Operating Characteristic Curve
SD: Standard Deviation
SBP: Systolic Blood Pressure
SMI: Skeletal Muscle Mass Index
TC: Total Cholesterol
TG: Triglycerides
T: Tertiles
VIF: Variance Inflation Factor
VFA: Visceral Fat Area
WC: Waist Circumference
