# Supplementary material for: Phase angle as a predictor of prolonged length of hospital stay and adverse outcomes in elderly medical inpatients: a retrospective cohort study
Source: Front Nutr. 2025 Aug 7;12:1623983. doi: 10.3389/fnut.2025.1623983 (PMC12367514; doi:10.3389/fnut.2025.1623983)
Supplement: Supplementary file 1 [file Supplementary_file_1.docx]

Supplementary Material

# Table S1 Schoenfeld residuals test for the proportional hazards assumption

| **Variables** | ***χ*^2^** | **df** | ***P*-value** |
| --- | --- | --- | --- |
| PhA | 1.311 | 1 | 0.25 |
| Age | 0.673 | 1 | 0.41 |
| Gender | 2.441 | 1 | 0.12 |
| Polypharmacy | 2.559 | 1 | 0.11 |
| Malnutrition | 0.513 | 1 | 0.47 |
| Functional trajectory | 0.324 | 1 | 0.57 |
| Disability | 1.851 | 3 | 0.60 |
| eGFR | 0.434 | 1 | 0.51 |
| NT-proBNP | 0.513 | 1 | 0.47 |
| Global test | 10.652 | 11 | 0.47 |

All *p*-values > 0.05 indicate no violation of proportional hazards assumption. BMI, Body Mass Index; eGFR, Estimated Glomerular Filtration Rate; df, degrees of freedom; PhA, Phase Angle; *χ*^2^, chi-square.

# Table S2 Results of collinearity analysis

| **Variables** | **VIF** | |
| --- | --- | --- |
|  | **Continuous PhA** | **PhA dichotomized by ROC cutoff** |
| PhA (form specified) | 2.016 | 1.521 |
| Age | 1.833 | 1.601 |
| Gender | 1.154 | 1.060 |
| Polypharmacy | 1.179 | 1.155 |
| Malnutrition | 1.242 | 1.234 |
| Functional trajectory | 1.317 | 1.316 |
| Disability | 1.748 | 1.715 |
| eGFR | 1.123 | 1.120 |
| NT-proBNP | 1.404 | 1.386 |

BMI, Body Mass Index; eGFR, Estimated Glomerular Filtration Rate; PhA, Phase Angle; ROC, Receiver Operating Characteristic Curve; VIF, Variance inflation factors.

# Table S3 Population baseline characteristics based on the composite adverse outcomes within one year

| **Variables** | **Total**  **（n = 218）** | **Composite endpoint**  **(n = 42)** | **Normal**  **(n = 176)** | ***P*-value** |
| --- | --- | --- | --- | --- |
| Age, years, M (Q₁, Q₃) | 75.50 (69.00, 81.00) | 79.00 (72.00, 85.75) | 74.00 (69.00, 80.00) | 0.027 |
| Male, n (%) | 120 (55.05) | 27（64.29） | 93（52.84） | 0.180 |
| Education, n (%) |  |  |  | 0.938 |
| Junior high school or lower | 76 (34.86) | 14 (33.33) | 62 (35.23) |  |
| High school or vocational high school | 83 (38.07) | 17 (40.48) | 66 (37.50) |  |
| Associate degree or higher | 59 (27.06) | 11 (26.19) | 48 (27.27) |  |
| Residence, n (%) |  |  |  | 0.082 |
| Rural | 46 (21.10) | 13 (30.95) | 33 (18.75) |  |
| Urban | 172 (78.90) | 29 (69.05) | 143 (81.25) |  |
| Marriage, n (%) |  |  |  | 0.434 |
| Married | 156 (71.56) | 28 (66.67) | 128 (72.73) |  |
| Others | 62 (28.44) | 14 (33.33) | 48 (27.27) |  |
| Health Insurance, n (%) |  |  |  | 0.082 |
| Employment-based Health Insurance | 172 (78.90) | 29 (69.05) | 143 (81.25) |  |
| Non-employment-based Health Insurance | 46 (21.10) | 13 (30.95) | 33 (18.75) |  |
| Cognitive impairment, n (%) | 29 (13.30) | 5 (11.90) | 24 (13.64) | 0.767 |
| Smoking, n (%) | 29 (13.30) | 8 (19.05) | 21 (11.93) | 0.222 |
| Drinking, n (%) | 25 (11.47) | 5 (11.90) | 20 (11.36) | 0.921 |
| Multimorbidity, M (Q₁, Q₃) | 2.00 (1.00, 3.00) | 2.00 (1.75, 3.00) | 2.00 (1.00, 3.00) | 0.226 |
| Polypharmacy, n (%) | 107 (49.08) | 32 (76.19) | 75 (42.61) | < 0.001 |
| Malnutrition, n (%) | 126 (57.80) | 29 (69.05) | 97 (55.11) | 0.100 |
| Functional trajectory, n (%) |  |  |  | 0.026 |
| Stable/Improved | 189 (86.70) | 32 (76.19) | 157 (89.20) |  |
| Decline/Death | 29 (13.30) | 10 (23.81) | 19 (10.80) |  |
| Disability, n (%) |  |  |  | < 0.001 |
| Normal | 109 (50.00) | 13 (30.95) | 96 (54.55) |  |
| Mild | 78 (35.78) | 11 (26.19) | 67 (38.07) |  |
| Moderate | 18 (8.26) | 12 (28.57) | 6 (3.41) |  |
| Severe | 13 (5.96) | 6 (14.29) | 7 (3.98) |  |
| SBP, mmHg, Mean ± SD | 136.57 ± 17.6 | 137.36 ± 17.80 | 136.38 ± 17.61 | 0.748 |
| DBP, mmHg, Mean ± SD | 73.35 ± 10.43 | 72.95 ± 9.71 | 73.45 ± 10.62 | 0.782 |
| FBG, mmol/L, M (Q₁, Q₃) | 5.16 (4.70, 6.24) | 4.90 (4.51, 6.22) | 5.20 (4.72, 6.25) | 0.205 |
| Albumin, g/L, M (Q₁, Q₃) | 37.35 (34.90, 39.60) | 36.20 (33.88, 39.13) | 37.60 (35.50, 39.78) | 0.022 |
| Prealbumin, mg/L, M (Q₁, Q₃) | 225.35 (183.93, 262.60) | 220.20 (165.50, 255.93) | 229.00 (195.00, 263.18) | 0.195 |
| eGFR, ml/min/1.73m², M (Q₁, Q₃) | 70.94 (60.16, 78.91) | 70.82 (55.77, 79.03) | 70.94 (61.89, 78.90) | 0.214 |
| NT-proBNP, (pg/mL), M ((Q₁, Q₃) | 108.00 (60.00, 236.00) | 246.00 (105.25, 560.75) | 100.00 (57.00, 181.00) | < 0.001 |
| TC, mmol/L, Mean ± SD | 4.43 ± 1.14 | 4.19 ± 1.20 | 4.49 ± 1.12 | 0.130 |
| TG, mmol/L, M (Q₁, Q₃) | 1.08 (0.81, 1.61) | 1.07 (0.80, 1.78) | 1.09 (0.82, 1.55) | 0.863 |
| LDL-C, mmol/L, M (Q₁, Q₃) | 2.41 ± 0.90 | 2.29 ± 0.84 | 2.43 ± 0.92 | 0.375 |
| HDL-C, mmol/L, M (Q₁, Q₃) | 1.37 (1.16, 1.60) | 1.22 (1.12, 1.55) | 1.40 (1.17, 1.63) | 0.034 |
| BMI, kg/m^2^, Mean ± SD | 24.44 ± 3.27 | 24.07 ± 3.27 | 24.53 ± 3.28 | 0.419 |
| SMI, kg/m^2^, Mean ± SD | 7.60 ± 1.18 | 7.45 ± 1.41 | 7.64 ± 1.12 | 0.363 |
| WC, cm, Mean ± SD | 80.10 ± 10.52 | 79.89 ± 12.14 | 80.16 ± 10.13 | 0.882 |
| VFA, cm^2^, M (Q₁, Q₃) | 77.75 (57.18, 104.15) | 77.80 (52.88, 118.68) | 77.55 (58.35, 103.88) | 0.866 |
| PhA, °, Mean ± SD | 4.73 ± 0.93 | 4.10 ± 0.89 | 4.89 ± 0.88 | < 0.001 |

BMI, Body Mass Index; DBP, Diastolic Blood Pressure; eGFR, Estimated Glomerular Filtration Rate; FBG, Fasting Blood Glucose; HDL-C, High-Density Lipoprotein Cholesterol; LDL-C, Low-Density Lipoprotein Cholesterol; PhA, Phase Angle; SBP, Systolic Blood Pressure; SMI, Skeletal Muscle Mass Index; TC, Total Cholesterol; TG, Triglycerides; VFA, Visceral Fat Area; WC, Waist Circumference. M, Median; Q₁, 1st Quartile; Q₃, 3st Quartile; SD, Standard Deviation.

# Table S4 Clinical characteristics of the study population by the cutoff value of the PhA

| **Variables** | **Total**  **(n = 218)** | **Low PhA**  **(n = 73)** | **Normal PhA**  **(n = 145)** | ***P*-value** |
| --- | --- | --- | --- | --- |
| Age, years, M (Q₁, Q₃) | 75.50 (69.00, 81.00) | 80.00 (76.00, 85.00) | 71.00 (68.00, 77.00) | < 0.001 |
| Male, n (%) | 120 (55.05) | 46 (63.01) | 74 (51.03) | 0.093 |
| Education, n (%) |  |  |  | 0.740 |
| Junior high school or lower | 76 (34.86) | 28 (38.36) | 48 (33.10) |  |
| High school or vocational high school | 83 (38.07) | 26 (35.62) | 57 (39.31) |  |
| Associate degree or higher | 59 (27.06) | 19 (26.03) | 40 (27.59) |  |
| Residence, n (%) |  |  |  | 0.834 |
| Rural | 46 (21.10) | 16 (21.92) | 30 (20.69) |  |
| Urban | 172 (78.90) | 57 (78.08) | 115 (79.31) |  |
| Marriage, n (%) |  |  |  | < 0.001 |
| Married | 156 (71.56) | 40 (54.79) | 116 (80.00) |  |
| Others | 62 (28.44) | 33 (45.21) | 29 (20.00) |  |
| Health Insurance, n (%) |  |  |  | 0.834 |
| Employment-based Health Insurance | 172 (78.90) | 57 (78.08) | 115 (79.31) |  |
| Non-employment-based Health Insurance | 46 (21.10) | 16 (21.92) | 30 (20.69) |  |
| Cognitive impairment, n (%) | 29 (13.30) | 12 (16.44) | 17 (11.72) | 0.333 |
| Smoking, n (%) | 29 (13.30) | 16 (21.92) | 13 (8.97) | 0.008 |
| Drinking, n (%) | 25 (11.47) | 10 (13.70) | 15 (10.34) | 0.463 |
| Multimorbidity, M (Q₁, Q₃) | 2.00 (1.00, 3.00) | 2.00 (1.00, 3.00) | 2.00 (1.00, 3.00) | 0.416 |
| Polypharmacy, n (%) | 107 (49.08) | 49 (67.12) | 58 (40.00) | < 0.001 |
| Malnutrition, n (%) | 126 (57.80) | 59 (80.82) | 67 (46.21) | < 0.001 |
| Functional trajectory, n (%) |  |  |  | < 0.001 |
| Stable/Improved | 189 (86.70) | 55 (75.34) | 134 (92.41) |  |
| Decline/Death | 29 (13.30) | 18 (24.66) | 11 (7.59) |  |
| Disability, n (%) |  |  |  | < 0.001 |
| Normal | 109 (50.00) | 20 (27.40) | 89 (61.38) |  |
| Mild | 78 (35.78) | 30 (41.10) | 48 (33.10) |  |
| Moderate | 18 (8.26) | 13 (17.81) | 5 (3.45) |  |
| Severe | 13 (5.96) | 10 (13.70) | 3 (2.07) |  |
| SBP, mmHg, Mean ± SD | 136.57 ± 17.6 | 137.90 ± 18.39 | 135.90 ± 17.23 | 0.428 |
| DBP, mmHg, Mean ± SD | 73.35 ± 10.43 | 72.21 ± 11.90 | 73.93 ± 9.60 | 0.250 |
| FBG, mmol/L, M (Q₁, Q₃) | 5.16 (4.70, 6.24) | 4.99 (4.53, 6.31) | 5.22 (4.75, 6.22) | 0.114 |
| Albumin, g/L, Mean ± SD | 37.07 ± 3.86 | 34.92 ± 4.05 | 38.15 ± 3.28 | < 0.001 |
| Prealbumin, mg/L, M (Q₁, Q₃) | 225.35 (183.93, 262.60) | 210.00 (166.75, 240.55) | 234.4 (206.85, 266.10) | < 0.001 |
| eGFR, ml/min/1.73m², M (Q₁, Q₃) | 70.94 (60.16, 78.91) | 68.15 (55.80, 81.32) | 71.85 (63.30, 78.76) | 0.105 |
| NT-proBNP, (pg/mL), M (Q₁, Q₃) | 108.00 (60.00, 236.00) | 236.00 (121.00, 702.00) | 83.50 (50.50, 133.00) | < 0.001 |
| TC, mmol/L, Mean ± SD | 4.43 ± 1.14 | 4.19 ± 1.15 | 4.55 ± 1.12 | 0.027 |
| TG, mmol/L, M (Q₁, Q₃) | 1.08 (0.81, 1.61) | 1.11 (0.77, 1.70) | 1.07 (0.84, 1.53) | 0.987 |
| LDL-C, mmol/L, Mean ± SD | 2.41 ± 0.90 | 2.30 ± 0.97 | 2.46 ± 0.87 | 0.225 |
| HDL-C, mmol/L, Mean ± SD | 1.40 ± 0.32 | 1.34 ± 0.32 | 1.43 ± 0.32 | 0.054 |
| BMI, kg/m^2^, Mean ± SD | 24.44 ± 3.27 | 22.89 ± 3.57 | 25.22 ± 2.81 | < 0.001 |
| SMI, kg/m^2^, Mean ± SD | 7.60 ± 1.18 | 7.19 ± 1.30 | 7.81 ± 1.06 | 0.001 |
| WC, cm, Mean ± SD | 80.10 ± 10.52 | 78.50 ± 13.02 | 80.91 ± 8.94 | 0.157 |
| VFA, cm^2^, M (Q₁, Q₃) | 77.75 (57.18, 104.15) | 76.90 (52.15, 111.50) | 78.20 (58.40, 101.50) | 0.999 |

BMI, Body Mass Index; DBP, Diastolic Blood Pressure; eGFR, Estimated Glomerular Filtration Rate; FBG, Fasting Blood Glucose; HDL-C, High-Density Lipoprotein Cholesterol; LDL-C, Low-Density Lipoprotein Cholesterol; PhA, Phase Angle; SBP, Systolic Blood Pressure; SMI, Skeletal Muscle Mass Index; TC, Total Cholesterol; TG, Triglycerides; VFA, Visceral Fat Area; WC, Waist Circumference. M, Median; Q₁, 1st Quartile; Q₃, 3st Quartile; SD, Standard Deviation.

# Figure S1 ROC curve of PhA for predicting prolonged length of hospital stay

**
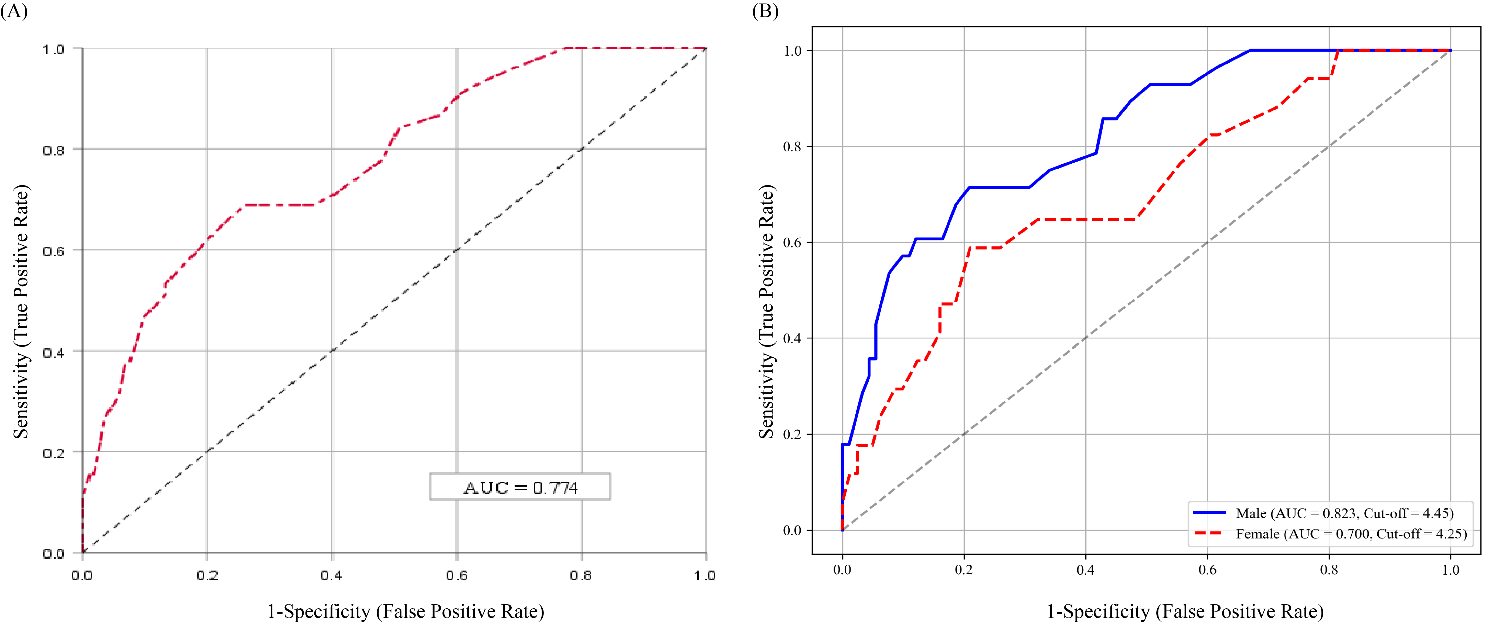
**

**Figure S1** ROC curve of PhA for predicting prolonged length of hospital stay. (A) ROC analysis based on the overall study population; (B) Sex-specific ROC analyses. AUC, Area Under the Curve; PhA, Phase Angle. The PhA cutoff values were 4.45° for men and 4.25° for women.
